# “I could do almost nothing without digital technology”: a qualitative exploration of adolescents’ perception of the risks and challenges of digital technology

**DOI:** 10.3389/fpsyg.2023.1237452

**Published:** 2023-12-12

**Authors:** Laura Bitto Urbanova, Andrea Madarasova Geckova, Zuzana Dankulincova Veselska, Silvia Capikova, Jana Holubcikova, Jitse P. van Dijk, Sijmen A. Reijneveld

**Affiliations:** ^1^Department of Health Psychology and Research Methodology, Faculty of Medicine, P. J. Safarik University Kosice, Košice, Slovakia; ^2^Graduate School Kosice Institute for Society and Health, Faculty of Medicine, P. J. Safarik University Kosice, Košice, Slovakia; ^3^Department of Community and Occupational Medicine, University Medical Center Groningen, University of Groningen, Groningen, Netherlands; ^4^Institute of Applied Psychology, Faculty of Social and Economic Sciences, Comenius University Bratislava, Bratislava, Slovakia; ^5^Institute of Social Medicine and Medical Ethics, Faculty of Medicine, Comenius University in Bratislava, Bratislava, Slovakia

**Keywords:** qualitative study, digital technology, subjective perceptions, risks, adolescents

## Abstract

**Background:**

The fast development of digital technology and of its use at even younger ages is significantly shaping the current generation of adolescents. This is leading to an almost unlimited accessibility that provides a large number of opportunities, but also to many challenges that adolescents have to face. The aim of our study was to explore the perceptions adolescents have of the risks of digital technology.

**Methods:**

We conducted online semi-structured interviews as a part of the international Health Behaviour in School-aged Children study. The sample consisted of 15 Slovak adolescents (mean age: 15.33; 20% boys). To analyse our data, we used consensual qualitative research and thematic analysis.

**Findings:**

Our findings confirmed that adolescents are aware of the risks associated with the use of digital technology. Regarding their specific types of the perceived risks, we identified four main themes: 1. dependence on the functionality of technology; 2. problematic control; 3. vulnerability in the virtual environment; 4. health risks. Adolescents thus want technology that is functional, safe and does not endanger their health.

**Conclusion:**

Despite the fact that adolescents know of the risks they may experience due the digital technology, they still use it. Preventive strategies should focus on functionality, safety and healthiness; furthermore, they should support the constant development of adolescents’ digital awareness and raising their awareness about effective and non-threating use of technology.

## Introduction

Without any doubt the digital world has had a significant impact on the everyday lives of young people. Today’s children are growing up surrounded by digital technologies that includes not only the Internet, but also devices, such as tablets, computers or smartwatches, that can also crucially affect their lives. Moreover, research shows that children start to use digital technology at an ever decreasing age (Hooft Graafland, 2018); e.g., more than 20% of children under 2 years old already know how to use digital technology, and this number increases with the children’s age (Kılıç et al., 2019; Ofcom, 2019). Digital devices have become the tool of first choice not only for them, but also for their parents, as they often use the smartphone as a way to save some time to do daily tasks or domestic chores in the case of the younger kids and to maintain a connection with the adolescents, to track them through the phone or to reach them in emergency situations (Kılıç et al., 2019; Bickham et al., 2021). Regarding the risks linked to the digital world, research shows that most parents do not think that their kids are exposed to inappropriate online behaviour or content by getting a smartphone (Bickham et al., 2021), and if so, that they would be able to deal with such an experience (Livingstone and Haddon, 2012). On the one hand, this might reflect parent’s belief that adolescents often have higher level of digital skills compared to themselves, which makes them more capable in their eyes to face the challenges linked to the digital world. On the other hand, it may also suggest a gap in parents’ awareness of the possible risks linked to the digital world.

Although digital technologies can indeed be truly supportive for adolescents’ life (Bitto Urbanova et al. 2023), even for those who are socially excluded, it can also have a detrimental impact (UNICEF, 2017). Evidence suggests that digital technology can be easily misused for risky behaviour that threatens adolescents’ safety, privacy or wellbeing, even with a wider reach (UNICEF, 2017). Moreover, its daily use increases the chances of adolescents being exposed to such experiences (Livingstone et al. 2011). The findings of the EU Kids Online study (2020) showed that more than 90% of 15–16-year-old adolescents face one or more risks during their use of digital technology (Smahel et al. 2020). This kind of experience can have detrimental consequences, especially in the period of adolescence, as it is characterised by the onset of several mental health diseases (Solmi et al., 2022). Thus, evidence is especially needed on adolescents’ point of view regarding the risks associated with digital technology.

Most research on digital technology explores online risks (Mascheroni, Olafsson, 2014; UNICEF, 2017; Smahel et al., 2020). These risks can be divided into following categories: content risks (adolescents as a recipient of aggressive, sexual content), contact risks (adolescents as participants/victims of cyberbullying, cyber dating abuse, online grooming, theft of personal information), and conduct risks (adolescents as a providers of cyberbullying, trolling, sexual harassment, gambling, creating pornographic material or providing advice linked to suicide or pro-anorexia) (Livingstone, Smith, 2014a; Livingstone et al., 2018). Using the types of online risks mentioned above, research has shown that content risks, especially cyberbullying, is the most prevalent online risk (30%) (Machimbarrena et al. 2018). However, the use of digital devices also has risks that are not necessarily related to the online space. Research indicates that its excessive use can cause problems of physical (digital eyestrain, musculoskeletal, sleeping issues, tiredness, obesity), mental (depression, anxiety, stress or isolation), social (relationships problems, decrease of the closeness and face-to-face conversation quality) or educational nature (decrease of academic performance) (Lajunen et al., 2007; Munezawa et al. 2011; Przybylski, Weinsten, 2013; Riehm et al., 2019; Rose et al., 2022). Until now, the topic of the risks associated with digital technology has been mostly explored in quantitative studies, not going in-depth on the experiences of adolescents.

To sum up, although there is evidence of the studies exploring the risks linked to the use of digital technologies, a major part of such studies is quantitative and mostly concerns the adult point of view. Qualitative research allows us to go more deeply into the backgrounds of the topic studied and to describe the adolescents’ personal experiences in relation with their local context. Moreover, in our previous study we identified various benefits associated with the use of digital technology based on adolescents' statements (Bitto Urbanova et al. 2023). Thus, the aim of this qualitative study was to also examine adolescents’ perspectives and feelings regarding the negative aspects associated with these devices. Such evidence can be very helpful in its next development and transformation into strategies and solutions that can be useful and safe for adolescents.

## Methods

### Design of the study

This qualitative study was performed as part of the international HBSC (Health Behaviour in School-aged Children) study, which is focused on the exploration of the health and health-related behaviour of adolescents with respect to their social context. We conducted this study in accordance with the criteria of consensual qualitative research (CQR) (Hill et al. 2005) and thematic analysis (Braun and Clarke, 2006).

The Ethics Committee of the Medical Faculty at the Pavol Jozef Safarik University in Kosice (19 N/2020) approved the study protocol. Moreover, the study was conducted according to the ethical standards laid down in the Declaration of Helsinki (World Medical Association, 1964).

### Study setting, sampling and participants

The target group for the interviews were Slovak students in the first year of secondary school, who were at the age of 14 to 16 years old. We obtained our study sample in several steps. First, the project researchers contacted the school administrators and only after receiving their approval with the study participation, did we contact the parents of potential respondents. If parents provided informed consent to their child’s participation, we then contacted the adolescents to inform them about the study. Moreover, they received information about the voluntary and confidential nature of their participation and about the possibility to withdraw from the study at any time. In the end, 15 adolescents in total (three boys; mean age = 15.3) from different types of secondary school in Kosice provided informed consent.

### Procedure and measures

First, we collected data on the adolescents’ sociodemographic characteristics (age, gender, size of the place of residence) and their level of Internet use. We measured the level of Internet use in adolescents by using an excessive Internet use scale that consists of five items with 4-point Likert-type responses (never or almost never, not very often, fairly often, very often) (Škařupová et al., 2015). This scale covers five dimensions of Internet addiction (IA), such as salience *(I have gone without eating and sleeping because of the Internet*); tolerance *(I have caught myself surfing when I am not really interested)*; withdrawal symptoms *(I have felt bothered when I cannot be on the Internet)*; conflict *(I have spent less time than I should with either family, friends or doing schoolwork because of the time I spent on the Internet)*; and relapse *(I have tried unsuccessfully to spend less time on the Internet)* (Šmahel & Blinka, 2012; Škařupová et al., 2015). In order to determine the presence of above-mentioned symptoms of internet addiction and to identify various levels of internet use (no excessive Internet use, excessive Internet use and Internet addiction), the responses were dichotomized as fairly often and very often vs. not very often, almost never, or never. Next, based on that, we categorised levels of Internet use, by using the “2 + 1 rule” proposed by Tao et al. (2010). That is, adolescents who showed salience and withdrawal symptoms, and one more symptom from those listed above were categorised as Internet addicts. As excessive Internet users we categorised adolescents who reported 1–3 symptoms from the whole list of symptoms with the exception of the combination that determined the label Internet addiction.

After the recording of these background characteristics, adolescents took part in semi-structured individual or group interviews, in total 9 interviews with 5 persons per group at maximum.

Previous research showed that almost 20% of adolescents prefer using internet to talk about their inner feelings or worries and over 35% of all adolescents aged 13 and 15 reported that they use mobile phones to feel better (Madarasová Gecková, Dankulincová, 2019). Moreover, several groups of adolescents have been shown to be at higher risk of become excessive internet users: adolescents with lower level of socioeconomic status or lower life satisfaction (Bitto Urbanova et al., 2019), who experienced some kind of discrimination (Bitto Urbanova et al., 2020), and adolescents who reported more emotional and behavioural problems (Bitto Urbanova et al. under the review). Based on this evidence and in line with the purpose of this qualitative study, we formulated the following research questions:

1. When and why do you start using your mobile phone or tablet, connect to the Internet or get online?
2. How does the Internet make your life easier? How does it help you?
3. In what way can the Internet be dangerous for people?
4. How do you know when time spent with your mobile phone, tablet or online is rather too long?
5. How should the mobile phone, tablet or Internet improve to serve you in the best way, becoming something that helps you, thanks to which you feel better or that helps you to get closer to your goals?

The interviews were conducted online using the Zoom software, as they occurred during the second wave of the Covid–19 pandemic (November 2020 – June 2021), and Slovak government regulations did not allow us to meet with respondents in face-to-face interaction. The interviews were conducted in the Slovak language; they lasted approximately 45–60 min and were video recorded. A trained professional who had working experience as psychologist for adolescents in an online counselling platform led each interview. The other members of the research team, who also participated in the interviews as silent observers, also have a background in psychology and one in sociology.

### Data handling and analyses

We first transcribed the interviews verbatim in the Slovak language. The transcriptions were checked to ensure the accuracy of transcription process and uploaded into MAXQDA, the software used for the coding and analysis process. The data were then coded by a team of coders following the rules of the Consensual Qualitative Research (CQR) methodology. The team of coders was formed by the lead investigator (AMG), both senior researchers (ZD, SC) and a junior researcher (LBU); all of them had undergone previous training in the CQR methodology. All coders watched the video recordings of interviews, read the transcripts and created codes for parts of interviews individually. Afterwards, all of the members met for cross-checking. They discussed the generated codes and interpretations until consensus was achieved.

The data obtained in the questionnaire mapping the sociodemographic characteristics and the level of internet use were used to describe our study sample. To identify the perceived risks of digital technology, each team member separately clustered the codes produced during the data handling into the themes and subthemes using the thematic analysis. After that, all team members met for cross-checking and discussed the created themes and subthemes until they agreed on the final thematic map.

## Results

### Background characteristics

Our sample comprised 15 adolescents. The majority of them were students from a secondary school with graduation. Regarding their level of Internet use, most of the respondents did not report symptoms of excessive Internet use or Internet addiction. Only four of them could be considered as excessive Internet users, one as addicted to the Internet (see Table 1).

**Table 1:** Background characteristics of the sample.

Gender	
Boys	3
Girls	12
Types of school	
Grammar school	3
Secondary school (with graduation)	11
Secondary school (apprenticeship certificate)	1
Age (Mean, SD)	15.33 (0.62)
Level of internet use (mean, SD)	8.33 (2.38)
No excessive internet use	10
Excessive internet use	4
Internet addiction	1

*Apprenticeship certificate – General Certificate of Secondary Education.

### Main themes

**Figure 1:**
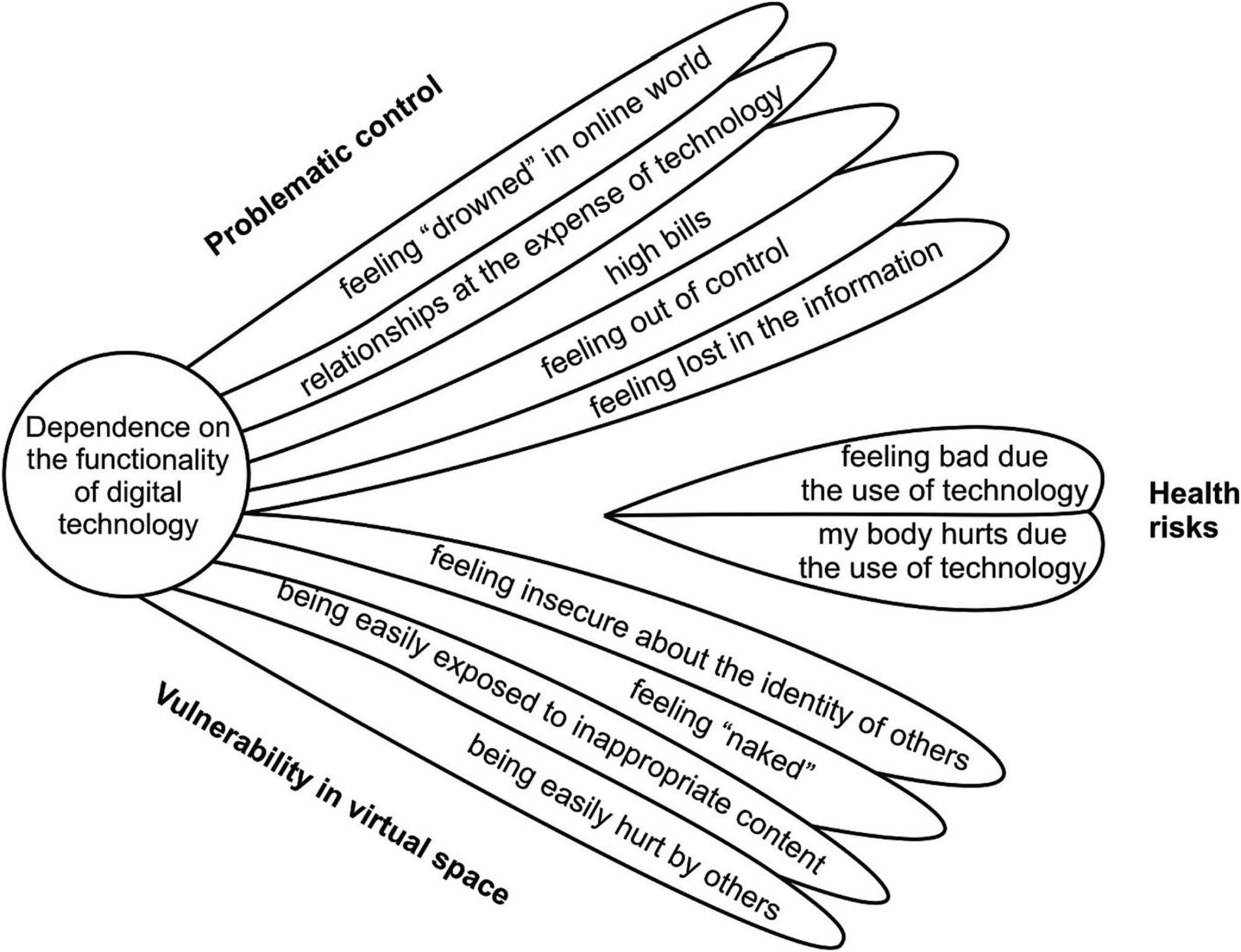
Model of themes and subthemes of perceived risks of digital technology and Internet use by adolescents.

We identified four main themes (Figure 1) regarding the risks and challenges that adolescents can face due to using digital technology: 1. Dependence on the functionality of technology; 2. Problematic control (*feeling “drowned” in the online world, relationships at the expense of technology, high bills, feeling out of control, feeling lost in information*); 3. Vulnerability in the virtual space (*feeling insecure about the identity of others, feeling “naked,” being easily exposed to inappropriate content, being easily hurt by others*); 4. Health risks (*my body hurts due the use of technology, feeling bad due the use of technology*). Table 2 presents some of the adolescents’ statements that led to the identification of the main themes.

**Table 2:** Selected participant quotes for the identified themes and subthemes on the risks of digital technology and the Internet.

Themes/Subthemes	Quotations
Dependence on technology	“It is kind funny for me, because when I move from room to room or I just go to the kitchen, I take the mobile phone with me, and I am walking… because I know that it will take a long time until I get to my phone again. I am the type of person, who needs to solve things in the moment, when I get some message, because you never know what kind of message is in your phone. “ “… for example, almost everything is online now, basically everything can be done online, you can do everything online, but if the Wi-Fi does not work, for example, we cannot do almost anything, and if the Wi-Fi cannot be fixed, you can lose everything that you have on Internet and I think that this can be pretty annoying, if it happens.”
Problematic control	
*Feeling out of control*	“… or for example, when I watch a movie, and then I want to see another one, and another, and another, and the fact is that I spend 2 h there by doing nothing…” “I try to spend less time on mobile phone, because when you start – you open the phone, and you just go, you are scrolling, and you just cannot stop doing it.”
*Feeling “drowned” in the online world*	“So, he/she does not have any other hobbies, aside from the Internet, and he/she does not even perceive reality, but he/she is always with the phone in hands, or watching TV or playing games, so this can be the disadvantage.” “… when I turn on the phone, for example, it is three o clock p.m., and then I actually immerse myself completely in the online world, and when I “wake up” again, it is already dark outside, for example, or that you can see the change in the time, and you did not realise it at all.”
*Relationships at the expense of technology*	“…Because, really, they can afford more on the Internet. Because they are showing their real face there, but if they come and talk with someone in face-to-face contact, it is harder for them.” “We spend more time with mobile phone or digital technology than with the people that we care about them.”
*High bills*	“When the Internet bill arrives.” “…and the negative part would be that you sit all the time with it and there is Wi-Fi connection every 300 metres, there, there and there, and then the bills and so on. You know, it does not grow in the garden, as we say.”
*Feeling lost in information*	“… on one webpage you can find the true and false information as well. So, it should be somehow marked there, that it may not be completely true, or not put it there at all. That there should be only the information that authors of the article are 100% sure that it is actually true.” “…that the webpages where the information can be found, are more verified. So, when I am working on some presentation, or I am looking for some information, I do not need to search for 20 different webpages about the same topic, but there will be one webpage with verified information…”
Vulnerability in virtual space	
*Feeling insecure about the identity of others*	“So, I think that especially social media can be dangerous, as you can be anonymous there. You can introduce yourself under a completely different name, and this can work for the criminals or bullies who provide cyberbullying, for example …” “For example, a lot of people are anonymous, somebody can say a bad thing about you or something like that. And it is anonymous. This person does not need to have a problem because of that. So, it is easy to write something bad to someone, and without any sanctions.”
*Feeling “naked”*	“…And people should be careful in working with the webpages, where they are required to share a lot of personal information and to not reveal a lot about themselves. It is a misuse of personal data, I think. And I also would not go to the insecure webpages.” “Sometimes we do not even have to share our personal information, but all we have to do is connect to some Wi-Fi, some kind of public Wi-Fi and those people already know everything through Wi-Fi, for example, where I live, how old I am; they just know all my information.”
*Being easily exposed to inappropriate content*	“Me, as a person who is interested in very strange things, I study conspiracy theories sometimes, and I get immerse so deeply in it, that it starts to scare me and I say to myself in that moment: Well, you should stop with it. Find out.” ” … when the kids are terribly at risk on the Internet, because they can easily get to banned webpages which they do not really need to see yet and it can also happen when they are playing games. …”
*Being easily hurt by others*	“…There is a Czech movie that came out now, where some girls who were over 18 years old, but they looked younger, had an artificial child’s room. I think it is called “In the network” and it was actually about the fact that those girls played 12–13 years old girls. That they were still kids, and those paedophiles wrote to them, so this is also dangerous from this point of view. Because a little child may not understand it, and this is dangerous and stressful.” “… so, it would not be so easy to get on someone’s profile. Because I have already received so many e-mails that somebody hacked my account. “
Health risks	
*My body hurts*	“… my back and neck start to hurt from sitting at computer, or my eyes because I was looking at computer for a long time.” “… or that I normally feel that I cannot be no longer at the Internet.”
*Feeling bad*	“…maybe the fact, that on the Internet people share a lot of information from their privacy, and then they envy each other, for example, when he/she puts there a picture of their new car and another one envies it.” “I think that, for example, that some relationships can be destroyed by social media, because it often happens that, for example, I am in some relationship, and jealousness plays a significant role in that relationship, and the partners argue because of social media …”

#### Dependence on the functionality of technology

Some adolescents expressed the risks and challenges associated with “dependence on the functionality of technology,” which is also the label for our first theme. Adolescents described various situations, when it was very important that their digital technology was working well, but it did not, such as when they needed to look for some information, to participate in online education or online leisure time activities or just to contact the people with whom they could not be in that moment. Moreover, adolescents proposed that it was not enough to have access to the Internet, but that in order to use it effectively, it is very important to have a digital skill on an adequate level. If not, it could easily lead to the exposure to other risks, such as the inappropriate protection of personal data.

The other problems associated with the functioning of digital technology mentioned by adolescents were old programs and applications that did not function well or that were not adapted to the needs of users. These could complicate their effective use. Adolescents perceived as a major problem the fact that many applications that could be very useful, for example, in the process of online education were not available in a language that users would understand. The other difficulty of many applications was that they were too complicated to be understood even when they using a language that the users could understand.

#### Problematic control

Regarding the theme “problematic control,” adolescents expressed several risks and challenges, such as the *feeling “drowned” in the online world, relationships at the expense of technology, high bills, feeling out of control* and *feeling lost in information.* Some adolescents found it difficult to stop using the technology when they were doing something interesting, such as watching a series, chatting with friends or playing online games, or even if they were just procrastinated by scrolling through posts on social media. It often happened to them that they *felt “drowned” in the online world*. To be specific, they were so immersed in some online activity that they did not realise how much time they had spent with the digital technology or the Internet until they looked through the window and it was already dark outside. Spending too much time with digital technology was also associated with another risk, i.e., *high bills* for the Internet and electricity.

Some adolescents reported that they sometimes *felt out of control* when using technology. They described the time spent on the Internet as a wasted or excessive, which could be used to do something more meaningful, such as preparation for school. However, they felt more relaxed if they had the digital technology at hand, even if they did not use it in that moment. The adolescents admitted that they used the digital technology or the Internet *at the expense of their relationships.* They described digital technology as a possible source of conflicts with their family members or friends. Adolescents admitted that spending too much time to the Internet could lead to neglecting relationships with people about whom they care. Moreover, Internet use could have a negative impact on their level of communication skills. The possibility to be in constant online contact often led to the loss of the depth of offline communication, or of the need to communicate offline at all.

Adolescents also mentioned information overload as another risk associated with the use of digital technology. They experienced many situations when they *felt lost in the information* available on the Internet, for example when they were looking for materials for a school project, and they encountered many webpages concerning the same or a similar topic. It was difficult for them to decide which webpage is the one with correct information. Even if they came across many webpages with misleading or disarranged information. The other risk mentioned by adolescents was a lack of information. They said that there were a lot of webpages focused on specific topics that were missing exact information, for example, a webpage presenting some kind of workout that did not explain the individual steps in the exercise and the possible consequences if the exercise was not done correctly.

#### Vulnerability in the virtual space

The adolescents mentioned several risks associated with the “vulnerability in the virtual space,” the label of our next theme. These risks were *feeling insecure about the identity of others, feeling “naked,” being easily exposed to inappropriate content* and *being easily hurt by others*. Adolescents reported that the users could often *feel “naked”* in the online world, as connecting only to public wi-fi could be perceived as direct way to make their personal information available to strangers. In addition, they said that users often published things on social media without considering the possible consequences. They forgot that their posts would still remain in the Internet database even after they deleted them.

The Internet was described by some adolescents as a dangerous place, where they could meet dangerous people. They said that they could *feel insecure about the identity of others*, as they did not know who was on the other side of the screen, as the anonymity on the Internet gave users the possibility to adapt their online identity to their own interests or to say what they thought without any sanctions. To be specific, on the one hand, the adolescents could find behind a profile someone who is truly polite and honest; on the other hand, there could be someone who did not have pure intentions and because of this, adolescents could *be easily hurt*. Those people could probably expose adolescents to some kind of harmful behaviour, such as cyberbullying, cyber grooming, misuse of personal data or blackmailing with photos or videos.

Another risk mentioned by adolescents was uncontrolled access to a variety of content on the Internet. In addition, they described insufficient age control as the biggest problem. Adolescents perceived that mainly children could *be easily exposed to inappropriate content* on the Internet, as the age control for many webpages was based only on children’s subjective statement. Adolescents did not even understand why some webpages, such as Facebook, were available also for kids who were 9 years old.

#### Health risks

The last theme, “health risks,” regarded the following subthemes: *my body hurts* and *feeling bad*. Adolescents reported different kinds of health problems associated with the use of digital technology, such as back pain, headache or eye pain. They said that in the moment when they felt that *their body hurt*, they knew that it was a sign that they had spent too long time on the Internet or at the screen of a computer and that there was a need for a change of activity. Moreover, they stated that digital technology also had a negative effect on their ability to concentrate, as it very often distracted them in performing different activities, such as during school preparation.

In addition, our respondents mentioned that sometimes they *felt bad* when they had to face the reality presented on the social media. They reported that their exposure to the posts of the people who were presenting their perfect life, in which they had everything that they ever dreamed of, and finding out that adolescents did not have anything like this in their own life, might lead to the feelings of sadness or envy.

## Discussion

We focused on the risks of digital technology according to adolescents’ subjective perceptions and experiences. We identified the following themes out of their statements: digital technology as a tool whose malfunctioning can complicate their life in various dimensions *(dependence on the functionality of technology);* a tool whose use sometimes makes them feel like they are drowning in the online world, out of control or lost in the information that it offers; and a tool that is used at the expense of relationships or can lead to high bills *(problematic control*). Moreover, it can expose them to people who make them feel insecure, because they feel naked or encounter inappropriate content or behaviour *(vulnerability in the virtual space)*; and it can cause bodily pain or negative feelings *(health risks)*. Our findings, compared to the studies based on an adult point of view, provide information on the broader context in which adolescents have to face to such risks (Núñez-Gómez et al., 2021). They show that adolescents are not only aware of the possible risks of digital technology, but they also have an idea of how technology should work so as not to endanger them.

### Main themes

#### Dependence on technology

We found that adolescents believed that they are unable to do almost nothing without digital technology and are thus highly dependent on it. This finding is in line with a study conducted by Kaspersky Lab (2015) showing that many people tend to use digital devices as the only place where they store their documents, contact information or memories. However, they often forgot about the risk of losing them. People perceive digital technology as a tool that can support their cognitive functioning. However, this kind of dependence can lead to a decrease in their own abilities (Uncapher et al., 2016) or creativity. Wilmer et al. (2017) showed that people’s trust in technology and unlimited access to their materials previously stored on digital devices reduces their need to save that information in their long-term memory. Thus, digital technology can be perceived as a tool which, on the one hand, can support our life in many different aspects thanks to its functions but which, on the other hand, has its own limits.

In our study adolescents also mentioned the risk of having insufficient digital skills what would limit their opportunities to benefit from the available applications and websites. This finding is in line with the EU Kids Online study showing that adolescents differ in their level of digital skills (Smahel et al., 2020). These differences can be due to factors such as low socioeconomic status, young age, insufficient digital education at school or insufficient parental mediation (Livingstone et al., 2019). However, the demands of todays digitised society refer to the crucial role that such skills play not only in using the opportunities of digital technology but also in determining the way adolescents would react upon exposure to digital risks (Smahel et al., 2020). Therefore, this subtheme raises the importance of supporting the development of children’s digital skills with the purpose of making the digital world safer and more accessible for all of them, as it can be a good way to reduce the inequality of opportunities among children from different backgrounds.

#### Problematic control

Our findings revealed that adolescents sometimes spent a huge amount of the time using digital technology, without even realising it. Moreover, they reported neglecting their duties and relationships or an increase in their bills. These findings are consistent with previous research showing that almost 15% of adolescents use digital technology at an excessive level (Filiz, 2015; Smahel et al., 2020; Lioupi et al., 2021). Our results can be explained in several ways. First, the features of digital technology can make it very attractive to adolescents; e.g., it represents an easy way to socialise, relax, gain information or just pass the time (Filiz, 2015; Jarman et al., 2021). However, an excessive use can indicate some difficulties in the offline world. In that case, technology can be perceived as a way to forget about offline problems for a moment. Second, digital technology also can help adolescents meet people with similar life conditions who can provide them the social support that they lack offline (Bitto Urbanova et al., 2020). All these motives may lead to the uncontrolled use of digital technology. However, this study was conducted during the second wave of Covid–19 pandemic in Slovakia, when people had to stay at home due the government regulations. Therefore, they had limited options to meet with friends, to entertain or attend the school what could turn in an excessive use of digital technology. Moreover, an increase in the prevalence of the symptoms linked to the excessive Internet use has been shown (Lobe et al., 2021; Paschke et al., 2021).

Based on our results, the problematic control linked to the use of technology can complicate their relationships. This can be explained as follows. First, it is not uncommon that adolescents use technology in someone’s company instead of paying the attention to face-to-face communication with that person. This behaviour can be caused by addiction to the Internet or smartphone or by the fear of missing out phenomenon (Chotpitayasunondh & Douglas, 2016). The communication partners can see that behaviour as a sign of disrespect and disinterest (Chotpitayasunondh & Douglas, 2018), which can cause conflicts. Second, thanks to social media, adolescents are exposed to other people’s posts, which often reflect only the highlights of their life. This can lead to social comparison with and unrealistic expectations of their partners and family (Lenhart, 2015). Third, as adolescents’ offline and online relationships are interconnected, the boundaries between these are often unclear, especially regarding the rules of that interaction. Therefore, the content of the online communication can be sometimes disclosed to other peers, which can cause distrust in relationships or social isolation (Abbasi and Alghamdi, 2017). To sum up, digital technology can be a very useful tool in developing social ties, if it is used with care and respect.

Adolescents also reported that sometimes they feel lost in the information available on the Internet. Although it is a great benefit that digital technology offers a huge amount of information, users sometimes find it difficult to work with it not only because of its high quantity, but also because of questionable quality. Therefore, its proper use requires the ability to verify its quality that not all users have. These people have to put in a lot of effort to obtain effective information and this is often accompanied by negative feelings, such as information anxiety (Naveed and Anwar, 2020). Moreover, evidence suggests that people who are more vulnerable to information overload are relatively unable to read effectively from computer screens (Chen et al.,2011). Thus, our results imply a need for development of the information management skills, and the school environment can play can play a key role in this.

#### Vulnerability in the virtual space

Adolescents reported several risks that reflect their vulnerability in the virtual space. Moreover, research shows that people who are vulnerable offline tend to spend more time with technology, which further increases their chance of experiencing online risks (El Asam and Katz, 2018; Katz, El Asam, 2019; El-Asam et al., 2022).

Regarding privacy, our adolescents mentioned that they sometimes feel “naked” due to losing control over their online personal information. Our finding is in line with evidence showing that today’s adolescents tend to share more personal information than they did in the past, although they are aware of the risks associated with doing so (Krombholz et al., 2012; Madden et al., 2013). This can be explained as follows. Social media in their current form allow people to disclose or build their social ties through sharing their personal information (Feng, Xie, 2014). Thus, adolescents looking for new social interactions, might make their personal information more accessible for strangers. Moreover, Roger’s protection motivation theory (1983) in the online context suggests that the evaluation of its possible risks or benefits has a key role in online disclosure. Thus, adolescents tend to share their personal information if it will bring a lot of benefits (Youn, 2005). Other specific motives that may be behind the adolescents’ tendency to share more personal information can be their desire to present themselves in a more favourable way, to develop their relationships, to be trendy, to store the memories or to entertain (Lee et al., 2008; Waters and Ackerman, 2011). Social media, thanks to their properties, can bring users instant gratification, e.g., positive feedback from others. Therefore, they are willing to take the risk that their personal data can be misused.

Our results showed that adolescents sometimes feel insecure about the identity of other users. However, the research conducted by Krombholz et al. (2012) suggested that adolescents do not worry about the identity of others if their profiles on social media contain similar personal information, such as the same location of living, schools attended or people they know. This gives them the feeling that they know this person. Regarding the creation of a false identity in the digital world, there are several explanations of this behaviour. First, Roger’s protection motivation theory (1983) in the online context proposed that if adolescents figure out that their disclosure will bring them more risks than benefits, they tend to share false or incomplete personal information to protect themselves (Youn, 2005). Second, people with low self-esteem and a lack of social support in the offline world can create a false identity to gain acceptance or popularity (Gil-Or et al., 2015). Therefore, social media are places where they can potentially satisfy their unmet offline needs. Third, false profiles can also be used by some people to gather personal data of others which they could not achieve personally. These data can be subsequently misused for other types of online risky behaviour, as also mentioned by our adolescents.

Our adolescents reported that sometimes they can be easily exposed to inappropriate online behaviour or content. This finding is in line with previous research showing that social media present a platform where it is easier to attack the privacy of others, to behave in an inappropriate way, such as cyberbullying, cyber grooming, online theft and online fraud, or to be exposed to hate comments often motivated by the other users’ frustration or inferiority (Anderson, 2018; Smahel et al., 2020; Ballaschk et al., 2021). The EU Kids Online study (2019) showed that the prevalence of adolescents who have faced some of the previously mentioned experience varies between 7–39% (Smahel, 2020). Our finding on the exposure to the inappropriate behaviour can be explained by the fact that the digital world gives aggressors the opportunity to attract their victim more easily than in the offline world, as they can follow them even to the places where they felt safe before and to reach them anytime during the day (Patchin and Hinduja, 2006). In addition, such online public attacks can have a far- reaching impact not only due to the wider audience reached, but also by the possibility to store compromising materials for future use (Kowalski et al., 2014).

Regarding our finding on the adolescents’ exposure to inappropriate content, studies conducted by Livingstone and Gorzig (2014b) and Hollá (2020) showed that such experience is often greater in people who tend to look for a sensation and to take part in risky activities or who suffer from some psychological difficulties, as they tend to visit risky website. Ševcíková et al., 2015 proposed various situations when adolescents feel bothered by this kind of content, e.g., if the message presents extreme forms of sex or is inadequate for their age or stage in building romantic relationships. To sum up, adolescents’ exposure to online aggression or inappropriate content can have a detrimental impact on their lives; thus, it is important not only to look for ways to prevent the mentioned risks, but also to develop the skills to cope with them properly if adolescents do indeed face them.

#### Health risks

We found that adolescents’ long exposure to digital technology may lead to the several health problems, such as pain symptoms in different parts of their body (neck, back, head or eyes), fatigue or problems with concentration, which confirms previous findings (Lanaj et al., 2014; Stothart et al., 2015; Harbard et al., 2016; Camacho et al., 2018; Scott and Woods, 2018; Khan and Ambati, 2022). However, a study conducted by Khan and Ambati (2022) revealed that digital technology in itself is not detrimental, but that other factors that can play a critical role, such as the posture of adolescent’s body when using it in the case of musculoskeletal problems. Moreover, evidence suggests that an adolescent’s sensitivity to the signals produced by digital devices is still increasing. This may negatively affect the adolescent’s performance in attention-demanding tasks (Moisala et al., 2016), as people have only a limited capacity to concentrate properly on various tasks performed at the same time (Stothart et al., 2015). The problems regarding concentration can also be associated with fatigue, another risk mentioned by adolescents, which can be caused by their exposure to digital technology right before the bedtime (Van den Bulck, 2007) and the associated sleeping problems (reduced ability to fall asleep, poorer and shorter sleep) (Cain and Gradisar, 2010; Harbard et al., 2016; Orzech et al., 2016; Scott and Woods, 2018). People’s need to be alert and constantly connected to the digital world, even at night, can be explained by their fear of missing something important not only regarding their friends on social media (fear of missing out), but also by the video games that they play for hours during the day (Scott and Woods, 2018).

Additionally, our adolescents mentioned that being exposed to people’s reality as presented on social media and its comparison to their own life can lead to feelings of sadness and of jealousy. Previous research has shown that only around 20% of adolescents reported negative emotions regarding presented posts (Lenhart, 2015). The study conducted by Appel et al. (2015) proposed that people who reported depressive symptoms tend to experience the situational envy regarding the posts presented by attractive profiles. The truth is that depressed individuals often turn to social media to get support, self-acceptance or interactions confirming their self-worth (Lin et al., 2016). However, their passive use and exposure to super positive posts reflecting only the highlights of someone’s life can lead to social comparison or the feelings of envy (Krasnova et al., 2013). Moreover, the theory on emotional contagion suggests that the strong tie between poster and reader leads to a greater chance of sharing the same emotions between them or experiencing only benign envy regarding the presented online reality (Lin and Uts, 2015). Therefore, following personally unknown people, such as social media influencers, and exposure to their seemingly perfect life can cause a lead to the negative emotions in users (Van de Ven et al., 2012).

#### Strengths and limitations

Our study has a several strengths; the first one is the qualitative design, which allows us to talk about the opinions and experience of adolescents related to the use of digital technology based on their own statements, which were video-recorded, transcribed verbatim and reviewed by all the team members. The second strength regards our use of the consensual qualitative research methodology, which limited the potential impact of the subjective perspectives of the researchers, as these had to achieve a consensus related to codes used for the analysed data. Third, this study adds to our findings on the benefits associated with digital technologies coming from previous study (Bitto Urbanova et al. 2023) allowing us to provide more balanced view of adolescents’ use of these devices.

A possible limitation is the small size of the sample and homogenous representation of the target group. Due the restrictions proposed by Slovak government during the second wave of Covid-19 pandemic in Slovakia we had to use an online platform to talk with them instead of face-to-face contact. This fact could affect the composition of our sample, since is more difficult to gain students to a study sample via the internet than in face-to-face contact at school, as due the internet they have more freedom in deciding if they want to participate in such interview or not. However, we reached saturation before we stopped the inclusion of new participants. Moreover, replication of this study in a more heterogeneous sample regarding gender and type of school will beneficial to confirm our findings. Another limitation is that in some of our interviews we included multiple adolescents, what might affect the way how they answered our questions. We took several measures to prevent this kind of bias; i.e., using open-ended questions to support them in providing answers based on their subjective perspective, and letting the interviews to be led by a moderator with much experience in working with adolescents. This moderator provided respondents with a friendly and pleasant atmosphere. He conducted various approaches to establish rapport with them, such as the use of humour, self-disclosure, and making displays of respect. Moreover, using multiple respondents in one interview also yielded us more perspectives regarding our research questions.

#### Implications

We found that adolescents are aware of the various risks related to the use of digital technology. Nevertheless, they still use it and expose themselves to the potential risks associated with it. However, our findings show that they have clear ideas about the way digital technology should work to be beneficial for them. They are calling for technology that is functional and safe. Moreover, the evolution of technology goes hand in hand with new, emerging risks; thus, adolescents’ digital skills need to be constantly developed. The key role in this process is played by their natural environment, consisting not only of their parents or friends, but also of teachers. However, this requires the huge digital transformation of schools and children’s education. An alternative option can be the development of more effective safety settings that will help adolescents maintain their digital privacy.

Our study also identified several health problems caused by the use of digital technology. This finding shows the importance of balance between the time spent with technology and the time dedicated to activities that can support adolescents’ health. Preventive strategies focused on raising adolescents’ awareness about effective and non-threating use of technology can be key here. Moreover, since life without technology is no longer possible, preventive health campaigns are needed, dedicated to the best practices for safe cohabitation with them, e.g., proper posture when using digital technology, ways of improving sleeping quality (turning off digital devices near bedtime, changing room light, perceiving the indicators of approaching bedtime). To sum up, although our study identified several risks associated with digital technology, its constant development implies a need for further research of new challenges or phenomenon associated with them, such as the fear of missing out, phantom phone signals, cyberchondria etc., as they can have a detrimental impact on the life of adolescents and their healthy transition to adulthood.

## Conclusion

The findings of our study showed that our adolescents are aware of the risks that they can experience during the use of digital technology. Although digital technology can be very helpful for many people, they need to be careful while using it, and this holds strongly for adolescents. A qualitative research strategy can be beneficial to explore future changes in the studied phenomena and suggest the way society should response to it.

## Data availability statement

The raw data supporting the conclusions of this article will be made available by the authors, without undue reservation.

## Ethics statement

The studies involving humans were approved by The Ethics Committee of the Medical Faculty at the Pavol Jozef Safarik University in Kosice (19 N/2020). The studies were conducted in accordance with the local legislation and institutional requirements. Written informed consent for participation in this study was provided by the participants’ legal guardians/next of kin.

## Author contributions

LBU, AMG, and ZDV, and SC participated in the design and coordination of the study, data collection, analysis, and interpretation of the data. LBU conducted literature searches, provided summaries of previous research, and drafted the initial manuscript. AMG, ZDV, SC, JH, JD, and SR provided supervision and contributed with their comments to the manuscript. All authors contributed to the article and approved the submitted version.
